# Quality improvement strategies in trauma care: review and proposal of 31 novel quality indicators

**DOI:** 10.5694/mja2.51699

**Published:** 2022-09-11

**Authors:** Joshua G Kovoor, Jonathan Henry W Jacobsen, Zsolt J Balogh, for the Trauma Care Verification and Quality Improvement Writing Group

**Affiliations:** ^1^ Australian Safety and Efficacy Register of New Interventional Procedures – Surgical Royal Australasian College of Surgeons Adelaide SA; ^2^ University of Adelaide Adelaide SA; ^3^ Queen Elizabeth Hospital Adelaide SA; ^4^ John Hunter Hospital Newcastle NSW; ^5^ University of Newcastle Newcastle NSW

**Keywords:** Traumatology, Trauma surgery, Accidents, traffic, Accidents, Accreditation, Critical care

Trauma care verification relies on benchmarking, and it has recently moved towards verification of trauma systems rather than individual centres

Traumatic injuries are a leading cause of mortality, and can be challenging to treat, particularly in situations of polytrauma.^1^ In Australia, traumatic injuries resulted in 532 500 hospitalisations in 2017–2018, and in 2015, they accounted for 7% of long term health conditions and were responsible for $8.9 billion of health expenditure.^2^ Initiatives to improve trauma care quality can be delineated into clinician‐led activities, such as morbidity and mortality meetings, and organisational initiatives, such as benchmarking.^3^, ^4^ Across Australia and New Zealand (Aotearoa), key organisational quality improvement initiatives include the Australia New Zealand Trauma Registry (ATR) and the Australian and New Zealand Trauma Care Verification Program (TCVP). This perspective aims to highlight the utilisation, barriers, facilitators and future directions of the ATR and the TCVP. Data were derived from a systematic search of PubMed (13 April 2021), and the expert opinion of a working group of trauma professionals (CSP, GC, MCR, RO, ZJB) was used to develop the novel quality indicators suitable for the Australian and New Zealand practice that are proposed.

## Trauma care verification in Australia and New Zealand

The TCVP is an important quality improvement initiative led by Royal Australasian College of Surgeons (RACS), with active participation from the Australasian College for Emergency Medicine, the Australian and New Zealand College of Anaesthetists, the College of Intensive Care Medicine of Australia and New Zealand, and the Australasian Trauma Society. It is a peer review‐based benchmarking process (Box 1) and participation is voluntary. To date, 40 sites have undergone verification, including the entire New Zealand trauma system (Box 2). The New South Wales Institute of Trauma and Injury Management recommended that all trauma services in the state undergo verification every 5 years,^5^ a position strongly supported by the RACS.

Box 1 **Levels of trauma services with descriptors of trauma care provided^7^**

**Table:** 

Trauma service level	Description of services provided
Level I	Provides full spectrum of trauma care for the most critically injured patients Has consultant available 24/7 Provides high level research, education, and quality improvement activities
Level II	Provides comprehensive clinical care for severely injured patients Provides clinical care similar to a level I hospital, but the hospital does not necessarily undertake the same level of research and education activities
Level III	Provides high quality care to non‐major trauma patients Provides prompt assessment, resuscitation, emergency surgery and stabilisation of major trauma patients before referral to higher level trauma service
Level IV	Provides resuscitation and stabilisation of major trauma patients before transfer to higher level trauma service Requires support for transfer process

Box 2 **Map of trauma care verification sites in Australia and New Zealand (Aotearoa)**

**Figure:**
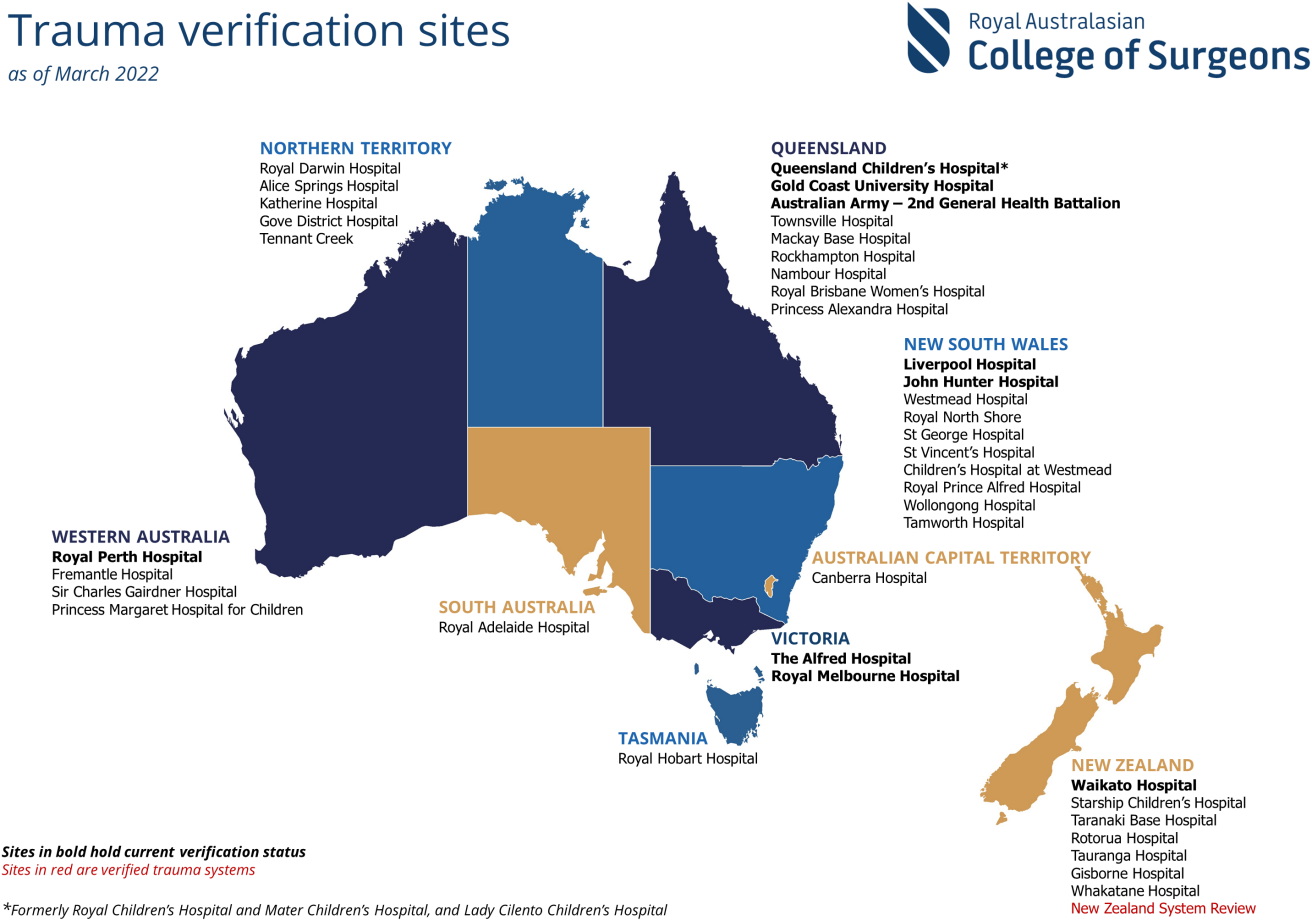
Source: Figure reproduced with permission from the Trauma Care Verification and Quality Improvement Writing Group, Royal Australasian College of Surgeons.

Trauma care verification relies on benchmarking, a process designed to improve care via the standardisation of clinical and administrative processes (Box 1).^6^ Trauma care verification in Australia and New Zealand benchmarks centres in accordance with the model resource criteria,^7^ and is a three‐stage process consisting of a pre‐review questionnaire, site visit, and the provision of feedback.^8^, ^9^ The pre‐review questionnaire is designed to appraise the institution’s trauma service in accordance with the model resource criteria and identify areas of focus for the site visit. It is ideally sent to the institution 6 months before the visit.^9^ The site visit is performed by a multidisciplinary team of trauma experts over 2 days. Case notes, guidelines and protocols are reviewed, and a tour following the path of the severely injured patient is performed.^7^ After the visit, a report is generated identifying the strengths, weakness and recommendations for improvement; this report is disseminated to hospital administrators and trauma service leaders about 3 months after the site visit.^9^ Recently, trauma verification has moved towards verification of trauma systems rather than individual centres.^10^ This is particularly relevant for geographically challenging countries such as Australia and New Zealand which also have a wide range of socio‐economic settings, and where time to definitive care may be impeded by distance and resources, making coordinated, system‐wide care crucial.^11^

## Effectiveness of trauma care verification

In an analysis of 109 American hospitals, verified level I trauma centres had lower mortality and complications and shorter intensive care unit and hospital stays compared with unverified centres.^12^ Verified centres were also more likely to comply with published guidelines.^13^ Trauma care verification also resulted in cost savings and greater investment in adjunct care.^14^ Following the implementation of several recommendations by the Trauma Care Verification Subcommittee when reviewing New Zealand, greater research support and funding was provided for the New Zealand Trauma Registry.^8^ Despite recent promising evidence, a meta‐analysis evaluating the effectiveness of trauma care verification noted limited generalisability of findings.^13^ The included studies were also exclusively performed in the United States and reflect the American College of Surgeons verification process. There is a need to conduct research evaluating trauma care verification in Australia and New Zealand, given the countries’ unique geographic, socio‐economic and health care system considerations.

## The Australia New Zealand Trauma Registry

The ATR was established in 2011 under the Australian Trauma Quality Improvement Program and currently collects data from 34 sites across Australia and New Zealand.^15^ The registry captures data from severely injured patients or patients who died following injury who were admitted to participating sites. It uses an agreed Bi‐National Trauma Minimum Dataset,^16^ which was developed to standardise trauma monitoring, facilitate international comparisons, and enable benchmarking of trauma systems in Australia and New Zealand.^17^ The ATR is committed to supporting hospitals in their quality improvement efforts. To achieve this, trauma centres are encouraged to submit complete data to the ATR to facilitate further research.

Contributing to national research is an essential criterion in the TCVP for level I trauma centres. However, institutions face many barriers when collecting and recording data; examples include a lack of funding and resources and an inability to capture pre‐hospital and post‐discharge data.^18^ Further, ten of the 67 fields lack comparability with international datasets, reducing comparisons and benchmarking to international standards.^17^ Clinicians want the ATR to capture additional patient outcomes, such as quality of life and long term function; data from patients with minor traumatic injuries; pre‐hospital and post‐discharge information; and data from non‐major trauma centres.^18^, ^19^

## Quality indicators

Quality indicators are evidence‐based metrics evaluating the quality of health care processes that influence patient outcomes.^20^ They facilitate the tracking and comparison of clinical performance with the purpose of identifying potential improvements and are routinely used for internal and external benchmarking. However, developing quality indicators for trauma care is challenging due to case heterogeneity. Many existing quality indicators are also limited in their utility as they are not evidence‐based and do not accurately identify process problems or capture the incremental improvements of modern quality improvement initiatives.^21^ There is a need to identify and incorporate new quality indicators of trauma management which may more accurately reflect clinical care performance. Ideally, new quality indicators should encompass structural, process and outcome elements of care, as reflected in Donabedian’s health care quality framework.^22^

There have been several attempts to develop standardised trauma care quality indicators internationally. A 2021 systematic review proposed a core set of 82 trauma quality indicators following international expert consensus.^23^ A 2020 evaluation identified 13 indicators from the German Trauma Register using a systematic review and input from a working group of trauma experts.^24^ The RACS Trauma Quality Improvement Subcommittee developed and defined eight binational process indicators to allow cross‐comparison and benchmarking of trauma care between sites and jurisdictions in Australia and New Zealand.^15^ The RACS model resource criteria require collection and reporting of all eight indicators by level I and II trauma centres.^7^ The identified indicators are mostly process and in‐hospital indicators. The bias towards these may relate to the ease of measurement and the perceived importance of in‐hospital care over pre‐hospital and post‐discharge care.^23^

## Proposal for novel quality indicators

Following discussion of the above systematic search of the international literature, a working group of trauma professionals (CSP, GC, MCR, RO, ZJB) developed 31 novel indicators for Australia and New Zealand trauma care (Box 3). These are not ranked in any particular order. Optimising anaesthetist and emergency medicine physician coverage in trauma centres will also benefit patients. The specific indicators were selected via a synthesis of literature review and expert opinion, and encompass a range of structural, process and outcome elements as per Donabedian’s health care quality framework,^22^ with time to interventions being a common theme among process indicators. This approach was used to enhance local and international applicability across trauma services, settings or resources. This may lead to increased adoption of the schema by trauma centres and to increased compliance with the quality indicators, without additional dedicated funding. However, they require validation to ensure optimal reliability and ideally, validation and cost–benefit analyses of the individual indicators. A key requirement of this validation assessment will be to ensure that, for those indicators that measure only certain aspects of global trauma system performance, the indicator goal correlates with improved patient outcomes. Importantly, several proposed indicators are already captured by the ATR and could thus be readily investigated. If the remaining indicators are valid and correlate with clinical improvement, the ATR should consider expanding their dataset to assess additional elements of care.

Box 3 Proposed quality indicators for measuring quality of trauma care in Australia and New Zealand (Aotearoa)*

**Table:** 

Category and subcategory	Quality indicator	Data can be obtained/calculated from the ATR
Structure		
In‐hospital	Blood alcohol screen within 6 hours	Yes
	Massive transfusion protocol	Yes
	Complete basic diagnostics available	No
	24‐Hour on‐site surgeon	No
	Trauma team activation	No
	Rate of organ donation	No
Pre‐hospital	Pre‐hospital time	Yes
	Pre‐hospital airway management in unconscious patient	Yes
	Pelvic binder in pelvic fracture	No
Process		
In‐hospital	Time in first facility if transferred	Yes
	Time to CT	Yes
	Time to first surgical intervention	Yes
	CO_2_ monitoring in intubated patients	No
	Time to first emergency surgery	Yes
	Time to craniotomy for severe TBI	Yes
	Time to surgery for haemorrhage control	Yes
	Time to soft tissue coverage of open tibia fractures	Yes
	Time to debridement and skeletal stabilisation of open long bone fractures	Yes
	Time to blood and products in shocked patients	No
	Time to rehabilitation from referral	Yes
	Discharge destination	Yes
Outcome		
In‐hospital	Incidence of nosocomial infection	No
	Pulmonary embolism, venous thromboembolism	Partial
	Length of emergency department stay	Yes
	Length of ICU stay	Yes
	Length of hospital stay	Yes
	Unplanned ICU readmission	No
	Unplanned return to operating room	No
	Unplanned hospital readmission rate related to the index trauma	No
	Mortality	Yes
After discharge	Functional outcome at time of discharge and at 6 months after injury	No

ATR = Australia New Zealand Trauma Registry; CO_2_ = carbon dioxide; CT = computed tomography; ICU = intensive care unit; TBI = traumatic brain injury.

* The proposed quality indicators for Australia and New Zealand were selected by a working group of trauma professionals (CSP, GC, MCR, RO, ZJB).

## Future directions

A recent survey of trauma professionals in Australia and New Zealand highlighted that trauma registries are underutilised.^19^ The recent agreement between the RACS, the NSW Agency for Clinical Innovation Institute of Trauma and Injury Management and the NSW State Insurance Regulatory Authority to verify all trauma centres in NSW, including regional centres, is a timely opportunity to generate Australian‐specific evidence. If trauma verification proves efficacious in NSW, it should serve as encouragement to the remaining jurisdictions to undertake the process. The ATR can potentially assist in the development of evidence‐based guidelines and policy and perform benchmarking and case reviews. Further, the ATR could link with other databases to track the long term outcomes.

Participation in quality improvement initiatives such as trauma verification and the ATR should be encouraged through additional government funding and policy support. Further research validating the 31 proposed quality indicators relevant to Australia and New Zealand trauma care is needed to determine their utility in practice.

## Open access

Open access publishing facilitated by The University of Newcastle, as part of the Wiley ‐ The University of Newcastle agreement via the Council of Australian University Librarians.

## Competing interests

No relevant disclosures.

## Provenance

Not commissioned; externally peer reviewed.
